# Human epidermal growth factor receptor 2 (*HER2*) gene amplification in non-muscle invasive urothelial bladder cancers: Identification of patients for targeted therapy

**DOI:** 10.1080/2090598X.2020.1814183

**Published:** 2020-09-02

**Authors:** Vinita Agrawal, Niharika Bharti, Rakesh Pandey

**Affiliations:** Department of Pathology, Sanjay Gandhi Postgraduate Institute of Medical Sciences, Lucknow, India

**Keywords:** Bladder carcinoma, non-invasive bladder carcinoma, *HER2* oncogene, fluorescent *in situ* hybridisation, immunohistochemistry, gene amplification

## Abstract

**Objectives:**

To evaluate human epidermal growth factor receptor 2 (HER2) protein overexpression by immunohistochemistry (IHC) and gene amplification by fluorescent *in situ* hybridisation (FISH) in urothelial non-muscle-invasive bladder carcinoma (NMIBC), as HER2 is a potential therapeutic target in muscle-invasive bladder carcinoma (MIBC) and HER2 expression and gene amplification in low/high-grade and pTa/pT1 NMIBC is not clear.

**Patients and methods:**

The study included 93 bladder cancers; 25 MIBC and 68 NMIBC (37 low- and 31 high-grade). All HER2 positive (3+) and equivocal (2+) cases were subjected to FISH using a HER2/CEN 17 dual-colour probe kit. IHC and FISH were scored as per the American Society of Clinical Oncology/College of American Pathologists (ASCO/CAP) 2013 Guidelines for breast cancers. Based on the number of signals/nuclei, amplification was categorised as low (≥6–10) and high-level (≥10).

**Results:**

HER2 2–3+ expression was seen in 29% of NMIBCs (10.8% low- and 51.6% high-grade). HER2 3+ expression was seen in high-grade NMIBC (nine of 31; 29%) and MIBC (nine of 25; 36%). In all, 87% of high-grade NMIBCs were lamina invasive (pT1). Gene amplification was found in 45% (eight of 18) of 3+ tumours. None of the HER2 2+ tumours showed gene amplification. IHC and FISH results were in closest agreement when ≥50% of tumour cells showed 3+ expressions. High-level amplification correlated with increased gene expression on reverse transcriptase-polymerase chain reaction. On multivariate analysis, lower  stage, grade, and HER2 expression significantly correlated with progression-free survival. HER2 3+ expression in NMIBC correlated significantly with time to recurrence and progression.

**Conclusion:**

Our present results show that HER2 FISH should not be performed for HER2 2 + and low-grade NMIBC. This contrasts with breast cancers where it is recommended for equivocal 2+ tumours. About 50% of HER2 3+ MIBC and high-grade NMIBC show *HER2* gene amplification and can be potential candidates for HER2-targeted therapy.

**Abbreviations:**

ASCO/CAP: American Society of Clinical Oncology/College of American Pathologists; DAB: 3,3ʹ-diaminobenzidine; FISH: fluorescent *in situ* hybridisation; HER2: human epidermal growth factor receptor 2; IHC: immunohistochemistry;(N)MIBC: (non-) muscle-invasive bladder carcinoma; MPUC: micropapillary variant of urothelial bladder cancer; PFS: progression-free survival; TURBT: transurethral resection of bladder tumour

## Introduction

Urinary bladder cancer ranks ninth in worldwide cancer incidence and is the seventh most common malignancy in males [1]. Clinically, non-muscle-invasive bladder cancers (NMIBCs) account for 70–75%, while the remaining 25–30% are muscle-invasive bladder cancers (MIBCs) or metastatic lesions at the time of initial presentation. Once invasive into muscle, bladder cancer has a poor prognosis and is responsible for most of the bladder cancer-related deaths. Patients with NMIBC have a better prognosis and they are treated by transurethral resection with adjuvant therapy, but they often present later with recurrence or invasive unresectable tumours.

With improvements in knowledge about its pathogenesis, the greater challenge is to identify biomarkers for clinical application. Studies have shown the potential prognostic value of markers including receptor tyrosine kinases such as fibroblast growth factor receptor 3 (FGFR3), epidermal growth factor receptor (EGFR), and ERBB2/human epidermal growth factor receptor 2 (HER2) in invasive bladder cancers [2].

There are data suggesting a role of HER2 in urothelial carcinoma and directed agents have entered clinical trials as potential therapeutic targets in locally advanced and metastatic bladder cancer [3,4]. Encouraging preclinical results with T-DM1 (ado-trastuzumab emtansine), consisting of the HER2 antibody trastuzumab conjugated with a cytotoxic agent, exemplifies a new potential treatment for HER2-positive bladder cancers. Studies have shown that HER2-positivity rates can differ between different populations [5]. An accurate assessment of HER2 status is important for proper patient selection. There are limited studies evaluating the correlation of HER2 2/3+ protein expression by immunohistochemistry (IHC) with *HER2* gene amplification in NMIBC [6,7]. The third (2004) and the fourth (2016) WHO Classification of tumours of the urothelial tract recommends classifying non-invasive papillary urothelial carcinoma as low- and high-grade [8].

To determine the utility and identify patients who could benefit from HER2-targeted therapy in low- and high-grade NMIBC, we analysed HER2 protein overexpression by IHC and gene amplification by fluorescent *in situ* hybridization (FISH) according to the American Society of Clinical Oncology/College of American Pathologists (ASCO/CAP) 2013 Guidelines for breast cancer.

## Patients and methods

Retrospective analysis of consecutive urothelial MIBCs (*n* = 25) and NMIBCs (*n* = 68) diagnosed on histology of transurethral resection of bladder tumour (TURBT) at the Department of Pathology, Sanjay Gandhi Postgraduate Institute of Medical Sciences (SGPGIMS), Lucknow, over a period of 3 years was performed. The TURBT tissues in which detrusor muscle was not included in the biopsy were excluded. Mesenchymal and non-urothelial bladder tumours were not included in the study. All patients with <2 years of follow-up were also excluded, except for patients who died of the disease in <2 years of diagnosis. The study was approved by the Institutional Ethics Committee.

Clinical and pathological data, including histological grade and extent of invasion of the tumour, were recorded. The histological grading was done according to the International Society of Urological Pathology (ISUP) WHO/ISUP (2004) system and the WHO 2016 classification of urothelial tract cancers [8].

Follow-up data included information about recurrence, time to recurrence, number of recurrences, progression documented on histology, time to progression, and disease-free survival.

### IHC

The IHC for HER2 was performed in all bladder cancers using rabbit anti-human c-erbB2 oncoprotein (SP3, Dako, Glostrup, Denmark) after heat-induced epitope retrieval. Polymer-based technique with 3,3ʹ-diaminobenzidine (DAB) as the chromogen was used for detection of the bound antibody. HER2 expression was scored according to ASCO 2013 HER2 test guidelines for immunohistochemical expression as 0–3+ for breast cancers [9]. HER2-positive (3+) and equivocal (2+) cases were subjected to FISH.

### FISH

FISH was performed in bladder cancers showing 2/3+ HER2 immunohistochemical protein expression using a HER2/CEN 17 dual-colour probe kit (ZytoLight Spec, Bremerhaven, Germany). Formalin-fixed paraffin-embedded tissue sections of 3–4 µm thickness were used and the area in the slide showing strongest expression for HER2 by IHC in the maximum number of tumour cells was marked for probe application.

A signal ratio of HER2 to chromosome 17 was recorded in a count of a minimum of 30 tumour cells and a ratio of ≥2 was regarded as *HER2* gene amplification. This was further categorised as low (≥6–10 *HER2* signals/nuclei) and high-level amplification (≥10 *HER2* signals/nuclei). Breast cancer with *HER2* amplification was taken as a positive control.

### Validation of FISH results by reverse transcriptase (RT)-PCR

*HER2* gene expression by quantitative RT-PCR was performed in 10 bladder tumours with HER2 3+ over-expression by IHC. The RNA was extracted from 10-µm thick formalin-fixed paraffin-embedded tissue sections using an RNA extraction kit from Chromous Biotech (Bangalore, India). Primers were designed and synthesised for human glyceraldehyde 3-phosphate dehydrogenase (GAPDH; housekeeping gene) and *HER2* gene. First-strand complementary DNA synthesis reactions were performed using Oligo dT primer. PCR was standardised to obtain amplification of the genes. All reactions were run in triplicate on an ABI Step-one real-time PCR machine. The output from the real-time software was analysed.

### Statistical analysis

Continuous variables between different groups of bladder carcinoma were compared using ANOVA and the categorical data was analysed by chi-square test. Survival analysis was done using Kaplan–Meier curves and differences in survival between groups were tested using the log-rank test. The survival end-point was taken as death due to disease or progression. Cox regression analysis was done to look for factors predicting time to recurrence and progression of bladder carcinoma in different groups. The Statistical Package for the Social Sciences (SPSS®), version 17 (SPSS Inc., Chicago, IL, USA), was used for statistical analysis and a *P* < 0.05 was considered as statistically significant.

## Results

The present study included 93 bladder tumours composed of 25 MIBCs and 68 NMIBCs. Of the 68 NMIBCs, 37 were low- and 31 were high-grade tumours. Lamina invasion (pT1) was found in most (27/31; 87%) of the high-grade non-invasive tumours, while most (27/37; 73%) of the low-grade non-invasive tumours were not lamina invasive (pTa). On follow-up, recurrence was seen in 29 (42.6%; 17 low-grade and 12 high-grade) patients with NMIBC. Progression in Stage/Grade was found in 16 (55%; seven low-grade and nine high-grade) of these 29 recurrences. Most of the patients were in their sixth and seventh decade of life, with a male predominance (*n* = 82; 88%). There was no significant difference in age at presentation or gender between the different groups.

**Figure 1.**
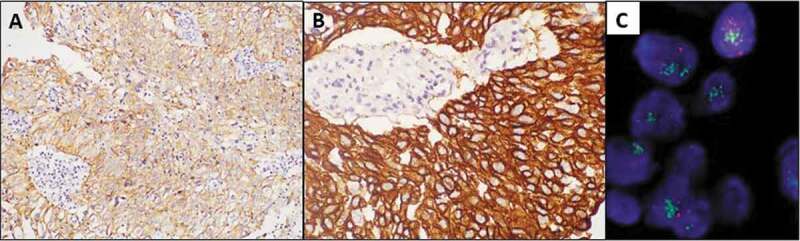
IHC for HER2 showing 2+ (A, DAB ×200) and 3+ (B, DAB ×400) expression in NMIBCs; *HER2* gene amplification by FISH was seen only in 3+ tumours (C; green signal HER2, red signal CEP17)

On IHC for HER2, 33% (31/93) of bladder cancers showed 2/3+ expression (Figure 1A and B). HER2 3+ expression was found in 19% (18/93) of the bladder cancers, comprised of 29% (nine of 31) high-grade NMIBCs and 36% (nine of 25) MIBCs (Table 1). All the high-grade NMIBCs showing HER2 3+ expression were lamina-invasive (pT1). The HER2 protein expression by IHC correlated with the stage (*P* < 0.05) and grade (*P* < 0.05) of bladder carcinoma.

**Table 1.** IHC expression of HER2 oncoprotein and correlation with *HER2* amplification by FISH according to the ASCO/CAP 2013 Guidelines on bladder urothelial carcinoma [8]

Category	HER2 (0, 1+), *n* (%)	HER2 (2+)			HER2 (3+)		
		Cases, *n* (%)	Amplification by FISH		Cases, *n* (%)	Amplification by FISH	
			Yes	No		Yes	No
LG-NMIBC (*n* = 37)	33 (89.2)	4 (10.8)	0	4	0	-	-
HG-NMIBC (*n* = 31)	15 (48.4)	7 (22.5)	0	7	9 (29)	4	5
MIBC (*n* = 25)	14 (56)	2 (8)	0	2	9 (36)	4	5

LG: low grade; HG: high grade.

All tumours with 2/3+ expression by IHC were subjected to FISH to evaluate for the presence of *HER2* gene amplification. None of the HER2 2+ tumours had *HER2* gene amplification by FISH. By contrast, 44.5% (eight of 18) of the tumours with HER2 3+ expression by IHC showed gene amplification (Figure 1C) and these comprised 8.6% (eight of 93) of all the bladder carcinomas.

HER2 3+ staining correlated with amplification by FISH (*P* < 0.05). High-level amplification seen in three tumours correlated with increased gene expression on RT-PCR.

The mean overall survival in low- and high-grade NMIBC, and MIBC was 135 and 99, and 61.7 months, respectively.

Kaplan–Meier analysis showed significantly (*P* < 0.05) better progression-free survival (PFS) in low-grade NMIBC followed by high-grade NMIBC and then MIBC. The log-rank test showed that PFS significantly correlated with stage, grade and HER2 3+ expression. In the NMIBC group, low-grade tumours had better patient survival than high-grade tumours. High-grade NMIBC had a significantly higher HER2 protein expression (*P* < 0.05), incidence of progression (*P* < 0.05), and lamina invasion (*P* < 0.05). However, there was no correlation with age at presentation, gender, recurrence, and history of prior recurrence.

As described by Chen *et al*. [6], IHC and FISH results were in closest agreement when over-expression was defined as 50% of tumour cells showing strong immunoreactivity.

## Discussion

Bladder carcinoma is characterised by the presence of two different clinical and prognostic subtypes: NMIBCs and MIBCs. MIBC has a poor outcome with common progression to metastasis, while NMIBC shows frequent recurrences.

HER2 is a member of the epidermal growth factor receptor family having tyrosine kinase activity. Dimerisation of the receptor results in the autophosphorylation of tyrosine residues within the cytoplasmic domain of the receptors and initiates a variety of signalling pathways leading to cell proliferation and tumorigenesis. The introduction of HER2-directed therapies has dramatically influenced the outcome of patients with HER2-positive breast and gastric/gastro-oesophageal cancers. There have been attempts to translate HER2 over-expression in MIBC into a therapeutic paradigm. However, the results have not been as promising as in breast cancer, possibly because HER2 over-expression in many urothelial carcinomas occurs without underlying genetic amplification [10]. Next-generation sequencing of advanced urothelial bladder cancers has revealed a diverse spectrum of actionable genomic alterations in 83% of cases, including HER2 mutations in 6% of tumours [11].

Most of the studies of HER2 expression in bladder carcinoma have been performed on MIBC and show varying expression ranging between 9% and 81% [12]. In a study on metastatic urothelial bladder carcinoma treated with platinum-based chemotherapy, Bellmunt *et al*. [5] reported that HER2 status varies in different populations. HER2 3+ staining and FISH amplification was seen in 22% of Spanish and 4% of Greek cohorts with no association with overall survival in univariate or multivariate analysis in any of the cohorts. In a study of 1005 invasive bladder cancers, Lae *et al*. [13] found lower HER2 2/3+ expression (9%) and gene amplification (5%). Other studies have reported *HER2* gene amplification in 10–12% of muscle-invasive and metastatic tumours [14,15]. In the present study from the Indian subcontinent, we found higher HER 2/3+ expression (44%), HER2 3+ expression (36%) and amplification (16%) in MIBCs. This variability could be due to the different populations studied, methods and cut-offs used, antibodies, as well as tumour heterogeneity and advanced stage at diagnosis.

MIBCs with a luminal molecular subtype have been shown to have a significantly higher rate of *HER2* alterations than those of the basal subtype, suggesting that HER2 activity is also associated with subtype status [16]. Authors have observed higher *HER2* amplification (15–40%) in micropapillary variant of urothelial bladder cancer (MPUC) compared with non-MPUC [17–19]. *HER2* over-expression has also been reported in plasmacytoid and lipid cell variants of urothelial bladder cancers [20,21].

However, reports of HER2 status in NMIBC are limited. A few studies have shown HER2 protein over-expression and gene amplification in 4–12% and 3–8% of NMIBCs, respectively [7,22,23]. Except for an occasional study, there is no clear data for *HER2* expression and amplification in the two groups namely, low- and high-grade NMIBC [6]. In the present study, we found HER2 3+ expression in 13% and gene amplification in 6% of NMIBCs. HER2 3+ expression and gene amplification was seen only in high-grade NMIBC. All of these tumours were lamina-invasive (pT1). Soria *et al*. [24], showed 2/3+ HER2 expression in 126 (36%) of 354 patients with MIBC and high-risk NMIBC refractory to intravesical chemotherapy/immunotherapy. In the present study, we found higher HER2 2/3+ expression (46%) in MIBC and high-grade NMIBC.

Studies have shown variable results regarding the prognostic significance of HER2 expression in urothelial carcinomas, Jimenez *et al*. [25] studied HER2 expression by IHC in MIBC and found that irrespective of primary or nodal involvement, it was not predictive of survival. In contrast, other studies have shown that HER2 immunoreactivity is significantly associated with shorter PFS and recurrence-free survival and disease-specific overall survival in urothelial carcinoma [3,6,7,26]. We also found a correlation of HER2 protein expression and gene amplification with PFS. Patients with MPUC having HER2 amplification had a worse cancer-specific survival than those who do not [18]. In non-invasive tumours, HER2 over-expression has been found to correlate with the incidence of recurrence and progression [27]. However, the authors considered HER2 expression as positive if membranous staining in >20% of tumour cells was seen, irrespective of the pattern and intensity, and they found no correlation of expression with a higher stage at presentation.

Variable degrees of correlation between HER2 protein over-expression and amplification have been reported in different studies. In a study of micropapillary and non-micropapillary urothelial carcinoma, HER2 IHC correlated with *HER2* amplification [17]. There are studies that did not identify a strong association between HER2 protein over-expression and gene amplification in high-grade invasive urothelial carcinomas [5,28]. Some studies have shown that HER2 amplification by FISH is seen only in HER2 3+ tumours with no amplification in 2+ tumours [13,29]. We also found *HER2* gene amplification by *in situ* hybridisation only in tumours showing 3+ protein expressions by IHC. A recent study found that when scored according to the 2013 ASCO/CAP Guidelines, 67% of HER2 3+ expressing advanced urothelial tumours were positive by FISH [30]. We found this correlation in 45% of HER2 3+ tumours. This difference could be attributed to the fact that our cohort includes both invasive and less aggressive non-invasive tumours.

Reports suggest that the closest agreement between IHC and FISH exists when over-expression is defined as 50% of tumour cells showing immunoreactivity [6]. We also observed that IHC and FISH results were in closest agreement when HER2 over-expression is defined as 50% of tumour cells showing strong complete membranous immunoreactivity.

A limitation of the present study was that none of our patients received HER2-targeted therapy and secondly, a relatively small sample size due to the exclusion of patients in whom follow-up data was <2 years. The approved HER2-targeted therapies for HER2-positive breast cancer include two antibodies (trastuzumab and pertuzumab), an antibody-drug conjugate (ado-trastuzumab emtansine), and a small molecule kinase inhibitor (lapatinib).

## Conclusion

Our present study shows that about one-half of non-invasive tumours show high-grade histology and HER2 3+ protein expression on IHC Therefore, confirmation of *HER2* gene amplification by FISH should be performed in all HER2 3+ high-grade NMIBC. However, FISH is not recommended for HER2 2+ and low-grade NMIBC. This contrasts with breast cancers where FISH is recommended for equivocal 2+ tumours.

HER2 IHC may thus be recommended as part of the management protocol in invasive and high-grade NMIBCs. HER2-targeted therapy may be a used as a treatment modality in such patients. Further studies are required to understand the molecular mechanisms, other than gene amplification that account for the high rates of HER2 protein over-expression in bladder cancers.
